# When Disasters Are the Norm: A 2050 Kidney Care Simulation. Report of a World Congress of Nephrology 2026 Spotlight Session

**DOI:** 10.1016/j.ekir.2026.107092

**Published:** 2026-09-25

**Authors:** Sayaka Ishigaki, Chia-Ter Chao, Ali K. Abu-Alfa, Serhan Tuglular, Marina Wainstein, Kyung Don Yoo, Sabine Karam, International Society of Nephrology Renal Disaster Preparedness Working Group (ISN RDPWG), Serhan Tuglular, Sabine Karam, Ali Abu-Alfa, Abdou Niang, Ahad Qayyum, Ali Düzova, Bronwyn Hayes, Christopher Atwater, Dirceu Reis Da Silva, Ionut Nistor, Kyung Don Yoo, Marina Wainstein, Sylvie Labbe

**Affiliations:** 1 Blood Purification Center, Hamamatsu University Hospital, Hamamatsu, Japan; 2 Division of Nephrology, Department of Internal Medicine, National Taiwan University Hospital and National Taiwan University College of Medicine, Taipei, Taiwan; 3 Graduate Institute of Toxicology, National Taiwan University College of Medicine, Taipei, Taiwan; 4 Graduate Institute of Medical Education and Bioethics, National Taiwan University College of Medicine, Taipei, Taiwan; 5 Division of Nephrology and Hypertension, Department of Internal Medicine, American University of Beirut, Beirut, Lebanon; 6 Department of Internal Medicine, Section of Nephrology, Yale University School of Medicine, New Haven, Connecticut, USA; 7 Marmara University, Istanbul, Turkey; 8 Faculty of Medicine, University of Queensland, Brisbane, Australia; 9 School of Medicine, University of Colorado Anschutz Medical Campus, Aurora, Colorado, USA; 10 Division of Nephrology, Department of Internal Medicine, Ulsan University Hospital, University of Ulsan College of Medicine, Ulsan, Republic of Korea; 11 Basic-Clinical Convergence Research Institute, University of Ulsan, Ulsan, Republic of Korea; 12 Department of Medical Science Convergence, Graduate School of Medical Science, University of Ulsan, Ulsan, Republic of Korea; 13 Division of Nephrology, University Health Network, Toronto, Ontario, Canada; 14 Division of Nephrology and Hypertension, Department of Medicine, University of Minnesota, Minneapolis, Minnesota, USA

**Keywords:** acute kidney injury, chronic kidney disease, climate change, dialysis, disaster preparedness, kidney failure

## Abstract

Recent disasters, including pandemics, armed conflicts, and extreme weather events, have highlighted the fragility of chronic dialysis systems and accelerated a broader shift in disaster nephrology from management and provision of dialysis for acute crush-related kidney injury toward resilience-oriented continuity of care planning for chronic kidney disease and kidney failure. At the World Congress of Nephrology 2026 held in Yokohama, Japan, the Spotlight Session entitled “When Disasters Are the Norm: A 2050 Kidney Care Simulation” explored future-oriented approaches to resilient kidney care. Two disaster scenarios and a future-oriented discussion were presented, involving water shortages, digital outages, and logistical disruption. The session discussed practical adaptation strategies including crisis-adapted dialysis protocols, decentralized and home-based dialysis models, low-technology backup systems, and regional coordination frameworks during resource-constrained conditions. The session also examined the potential future role of artificial intelligence (AI)-based forecasting systems which may support anticipatory dialysis preparedness through improved prediction of extreme weather events. Collectively, these discussions suggest that future disaster nephrology will increasingly require resilience-focused, decentralized, and operationally adaptable kidney care systems capable of sustaining continuity of care under conditions of recurrent disruption and infrastructure instability.

## Introduction

Climate change with an increasing frequency of extreme weather events, heat stress, floods, earthquakes, typhoons, tsunamis, is expected to substantially reshape the global burden and delivery of kidney care over the coming decades.^1^ In addition, other disasters such as pandemics and armed conflicts are no longer episodic shocks to health systems. They are also increasing in frequency, severity, and duration.^2^ Historically, disaster nephrology responses have largely focused on the immediate management of crush syndrome and provision of dialysis for acute kidney injury after mass-casualties events, particularly earthquakes.^3^, ^4^ However, more recent disasters such as pandemics,^5^, ^6^ and armed conflicts,^7^, ^8^ have reinforced concerns regarding vulnerability of chronic kidney care systems,^9^, ^10^, ^11^ including disruption of maintenance dialysis, medical supply chains, communication networks, trained personnel, and continuity of patient care.^12^ Patients receiving kidney replacement therapy are particularly vulnerable because traditional dialysis systems depend heavily on uninterrupted access to water, electricity, transportation systems, medical supply chains, communication networks, and trained personnel. Consequently, even relatively short disruptions in infrastructure may rapidly compromise life-sustaining care.^13^, ^14^ These experiences have reinforced the need for disaster preparedness in nephrology to move beyond acute emergency response toward resilience-oriented planning, decentralized care strategies, patient-centered preparedness, and international coordination frameworks.^10^, ^14^, ^15^

In this context, the International Society of Nephrology established the Renal Disaster Relief Task Force,^16^ which later evolved into the International Society of Nephrology Renal Disaster Preparedness Working Group, to strengthen global collaboration in disaster preparedness, response, and recovery for kidney patients.^17^ Building upon lessons learned from past disasters, the Renal Disaster Preparedness Working Group has increasingly expanded its focus from immediate disaster response toward broader discussions regarding future resilience of kidney care systems under conditions of climate instability, geopolitical uncertainty, and infrastructure vulnerability. Against this background, the World Congress of Nephrology 2026 Spotlight Session in Yokohama, Japan, “When Disasters Are the Norm: A 2050 Kidney Care Simulation,” explored future-oriented approaches to resilient kidney care through interactive disaster microscenarios. Using simulations involving water shortages, digital outages, and logistical disruption, the session aimed to provoke discussion regarding operational resilience, decentralized dialysis strategies, adaptive triage models, and future disaster governance frameworks for kidney care. The session particularly benefited from the presence of experts belonging to the Asia-Pacific region, the most disaster-prone region in the world, home to 75% of people affected globally.^18^

This report summarizes the 3 sections of the session. The first describes a water shortage scenario drawing on the Japanese earthquake experience. The second addresses a digital outage and logistical disruption scenario using a typhoon preparedness framework. The third considers future directions for resilient kidney care, including decentralized dialysis models and AI-assisted preparedness tools. To provide a consistent framework for interpreting these discussions, we use the terms preparedness, response, recovery, and resilience in a manner aligned with the World Health Organization Health Emergency and Disaster Risk Management Framework.^19^ Preparedness refers to measures taken before an event to develop the capacities needed for an effective response. Response comprises the immediate clinical and operational actions taken during a disruption, and recovery includes the restoration of services, evaluation of the response, and incorporation of lessons after the acute phase. Resilience refers to the broader capacity of patients, facilities, and health systems to anticipate, absorb, adapt to, and recover from disruption. Several measures discussed during the session, including regional communication networks, mutual-aid agreements, and home dialysis support, contribute to more than 1 phase and are therefore considered cross-cutting components of resilience.

## Methods

### Session Framework

The scenarios were developed by members of the International Society of Nephrology Renal Disaster Preparedness Working Group to prompt discussion of plausible threats to continuity of kidney care by 2050 and potential responses to them. The World Congress of Nephrology 2026 Spotlight Session panel included Sayaka Ishigaki (Japan), Chia-Ter Chao (Taiwan), and Kyung Don Yoo (Republic of Korea), each with expertise in disaster preparedness and response for dialysis and kidney care. Audience discussion and an informal show-of-hands poll were used to stimulate discussion, but the denominator was not recorded, and polling was not used to establish consensus or support recommendations. The statements in this report draw on lessons from previous disasters, views expressed by individual panelists and participants, and the authors’ subsequent synthesis of relevant literature and the session recording. No Delphi process, systematic evidence grading, or other formal consensus method was undertaken. Therefore, the report should not be interpreted as a clinical practice guideline or formal consensus statement.

#### Session 1: Scenario of Water Disruption

Japan has experienced several major earthquakes affecting dialysis care, including the Great Hanshin-Awaji Earthquake in 1995,^20^, ^21^ and the Great East Japan Earthquake in 2011.^22^ During these disasters, several hemodialysis facilities experienced severe disruption because of water shortages, power outages, and infrastructure damage. Most patients were able to receive dialysis treatment within 3 days, although some patients reportedly did not receive dialysis treatment for as long as 7 days during the Kobe Earthquake in 1995.^23^ Nevertheless, through nationwide cooperation, interfacility coordination, and patient transportation systems, dialysis treatment continuity was maintained.^22^, ^23^ These experiences emphasized the importance of preparedness, flexible dialysis strategies, and regional coordination during disasters. The following scenario, modeled on these real-world experiences, illustrates the clinical decision-making challenges that arise when water becomes the limiting resource.

##### Consider the Following Scenario

A major earthquake has occurred in a coastal region with a large dialysis population. Electricity remains partially available through backup generators, but the municipal water supply has been stopped. Your dialysis unit has enough water for only several hours, while dozens of patients are waiting for treatment. Under these conditions, maintaining routine dialysis schedules becomes impossible. Instead, health care providers must rapidly shift from standard dialysis care to crisis-adapted “survival dialysis” strategies.

In this report, “survival dialysis” refers to temporary, individualized, crisis-adapted dialysis delivered when usual resources are severely constrained, with the immediate aim of preventing or treating life-threatening complications until standard dialysis can be restored or the patient can be transferred. It is not a fixed shortened prescription and should not be applied uniformly. The clinical, operational, and procedural considerations that should inform this approach are summarized in Table 1.^10^, ^12^, ^24^, ^25^ Formal crisis allocation should be activated only after feasible measures to conserve resources, expand capacity, modify prescriptions individually, and redistribute patients regionally have been exhausted. When unavoidable, allocation should follow a preestablished, authorized triage process using transparent, equitable, and consistently applied clinical criteria, with documentation, oversight, or appeal where feasible, and safeguards against discrimination on nonclinical grounds (Table 1). Because patient acuity and available resources change, decisions should be reassessed regularly and usual standards restored as soon as conditions permit.^12^, ^24^

**Table 1:** Proposed framework for individualized crisis-adapted (survival) dialysis^a^

Domain	Assessment and safeguards
Working definition used in this report	Temporary, individualized dialysis delivered when usual resources are severely constrained, with the immediate aim of preventing or treating life-threatening complications until standard dialysis can be restored or the patient can be transferred.
1. Immediate dialysis threats	Severe or worsening hyperkalemia Severe volume overload Severe metabolic acidosis Uremic complications or rapidly worsening symptoms
2. Factors modifying whether treatment can be deferred or reduced	Residual kidney function and urine output, including responsiveness to diuretics Time and adequacy of the most recent dialysis treatment Vascular access Interdialytic weight gain and current volume status Potassium level and ability to use potassium binders Acid-base status Hemodynamic stability Comorbidities Anticipated time and access to the next treatment or a receiving facility
3. Operational context	Water, power, machines, staff, consumables, medications, transport, and receiving-facility capacity Expected duration and geographic scale of the disruption
4. Safeguards	Use the least restrictive contingency measure that can safely expand capacity Do not apply a fixed shortened prescription uniformly Reassess clinical status and resources repeatedly and restore usual care as soon as feasible Distinguish contingency prescription modification from formal crisis-allocation triage If formal allocation is required, use an authorized triage process with transparent and consistently applied criteria, documentation, appropriate oversight or appeal, and avoidance of discrimination based on disability, social worth, nationality, socioeconomic status, or other non-clinical characteristics

a This proposed framework represents an evidence-informed author synthesis and has not been prospectively validated as a disaster triage protocol.^10^, ^12^, ^24^, ^25^

Understanding water requirements is a critical component of disaster preparedness and response. A conventional hemodialysis session may require approximately 120 liters of dialysate. When reverse osmosis reject water is included, the total raw water requirement may amount to 200 to 500 liters per treatment depending on reverse osmosis recovery efficiency.^26^ Consequently, a dialysis unit treating 30 patients may require approximately 6000 liters of water per day. Awareness of these requirements is essential for realistic disaster planning. Green hemodialysis initiatives aim to reduce routine water, energy, and material consumption. However, lower baseline consumption alone does not ensure continuity of care when the water supply fails. In terms of preparedness, disaster resilience also requires additional measures, including protected emergency water storage, redundant power and water connections, alternative water sources that can be treated safely, and plans for rapid resupply. Lower baseline consumption may reduce demand during a disruption, but it does not substitute for storage, redundancy, or validated alternative sourcing.

In terms of response, several practical adaptation strategies can reduce water consumption while maintaining essential dialysis services. They include shortening dialysis sessions, reducing dialysate flow rates, and using isolated ultrafiltration when appropriate. In Japan, standard hemodialysis commonly uses a dialysate flow rate of approximately 500 ml/min. Reducing dialysate flow can decrease water consumption but also reduces small-solute clearance. The magnitude of this effect depends on the dialyzer, blood-flow rate, treatment duration, and patient characteristics. A recent systematic review and meta-analysis found that dialysate flow rate 300 ml/min was associated with a mean difference in spKt/V of −0.18 (95% confidence interval, −0.22 to −0.15) compared with 500 ml/min.^27^ Reduced dialysate flow rate may be considered on an individualized basis in emergency settings. If considered during a severe water shortage, it should be treated as a temporary, individualized contingency measure rather than an equivalent replacement for the usual prescription. Similarly, temporary shortening of treatment duration may also expand dialysis capacity during severe resource limitations but requires individualized assessment. Patients with both uncontrolled volume and potassium status may not be suitable for shortened treatment.^24^ Facility-level preparedness is important in that sense. In addition, dialysis centers should understand their own water storage capacity, backup systems, and recovery timelines. Some facilities maintain access to groundwater and on-site purification systems that can serve as alternative water sources during municipal supply interruption. Importantly, preparedness extends beyond equipment and infrastructure. Repeated drills and multidisciplinary communication are necessary to facilitate rapid decision-making during actual disasters. Staff members should understand how dialysis protocols may change during a crisis and how prescriptions should be individualized according to each patient’s clinical profile (Table 1).^10^, ^12^, ^24^, ^25^

The importance of adaptive dialysis strategies was clearly demonstrated during the Great East Japan Earthquake in 2011,^22^, ^28^, ^29^ which had a magnitude of 9.0, the largest earthquake recorded in the history of Japan. This was also a historic event for dialysis therapy because it was the first time that an earthquake of this magnitude occurred while dialysis treatments were actively being performed.^22^, ^29^ The disaster severely disrupted dialysis services across the affected region. In Miyagi Prefecture, 53 dialysis facilities were affected, and available dialysis bed capacity decreased to only approximately 14% of predisaster levels. Under these circumstances, a regional referral hospital assumed leadership by securing and concentrating the water supply at its facility, centralized dialysis services, and accepted patients from surrounding areas. Dialysis sessions were initially shortened to 2.5 hours, and the facility operated continuously for 24 hours per day and performed up to 8 dialysis cycles or shifts daily. More than 1000 dialysis sessions were reportedly provided within only 3 days after the disaster. Initially, treatment focused on maximizing patient coverage using shortened “survival dialysis” protocols. From the fourth day after the disaster, 3-hour dialysis sessions were implemented, and from the fifth day onward, treatment gradually returned toward standard 4-hour schedules.^22^, ^29^ These experiences demonstrated that flexibility and adaptation, rather than strict adherence to routine dialysis protocols, are essential during disaster response. In addition, after the Great East Japan Earthquake, a Japanese working group established by the Japanese Society for Dialysis Therapy, the Japanese Society of Nephrology, and the Japanese Association of Dialysis Physicians recommended that patient transfer outside the affected area should be considered when interruption of dialysis services exceeds 4 days or is expected to be further prolonged.^22^ Such patient transfer requires preparedness of interconnected infrastructure systems. Effective patient redistribution requires preestablished regional referral networks, clear communication pathways, and real-time situational awareness regarding dialysis capacity across neighboring facilities. These coordination mechanisms should be developed before disasters occur rather than during crisis response.

Taken together, this scenario and the described experience from Japan highlight an increasingly important challenge in disaster nephrology: how to continue dialysis care when water becomes the limiting resource. In such situations, the primary goal shifts from providing optimal dialysis to maintaining the greatest possible patient survival using limited resources. As climate change and extreme weather events become increasingly common, preparedness for water shortage will become an essential component of dialysis disaster planning worldwide.

#### Session 2: Scenario of Digital Outage and Logistic Delay

Although there has been a profound transformation and optimization of health care delivery through digitalization including disaster preparedness,^30^ vulnerabilities persist and are often abruptly unmasked during catastrophic events. Digital breakdown resulting from power outages, disruption of base stations, or cell tower failures may occur before, during, or after both natural and human-caused disasters. Such failures can compromise the digital infrastructure on which modern dialysis care depends, thereby hindering treatment for patients with kidney failure. In parallel, disasters may severely disrupt local geography, impairing transportation networks and compromising logistic efficiency. These challenges place nephrologists in a critical position, necessitating proactive planning, as emphasized in previous reports.^11^, ^31^

##### Consider the Following Scenario

You are the medical director of a major dialysis unit on an island nation in the Pacific Ocean. This region is frequently affected by typhoons, heavy rainfall, and earthquakes, all historically disrupting health care delivery. You learn that a severe typhoon is expected to reach your region within 1 week, with your unit situated near the projected trajectory. Concern arises regarding whether your dialysis unit can maintain operations. What actions should you take?

##### Devising a Locally Tailored Disaster Preparedness Plan

The panel present at the World Congress of Nephrology 2026 session, recommended developing a context-specific preparedness plan, tailored to the local geographic and sociocultural environment. Several guiding principles should be emphasized: prioritizing survival over normal operations, maximizing patient coverage over standard-quality dialysis delivery and low-technology over high-technology solutions. These principles reflect the realities of constrained resources during disasters and will be elaborated below. To structure preparedness efforts, the “5W1H” framework (why, when, which, who, where, and how) can be considered.

##### Why?

Preparedness begins with motivation: both patients and staff need to understand why disaster planning matters. Although increasing attention is directed toward climate and environmental resilience of kidney care,^32^ less focus is placed on the psychological preparedness and resilience of patients and health care personnel, who are vulnerable to psychological distress during disasters.^33^ Wellness and performance can be affected, hindering the accessibility, functionality and safety of health care systems.^34^ Early engagement with dialysis patients to codevelop preparedness strategies can improve emotional stability and adherence during crisis and recovery. For all unit members, investing effort in engaging and preparing them strengthens their readiness and confidence toward disaster preparedness. Regular drills annually or semiannually and structured educational programs can enhance preparedness, knowledge, and technical competency among unit members.^35^ Ultimately, elements of preparedness should address 4 components to achieve optimal outcomes: wellness, education, resources, and communication.^34^

##### When?

Timing is a critical determinant of preparedness effectiveness. In this scenario, weather forecasts provide an opportunity to delineate 2 key windows:

1. Predisaster window: This period allows for implementation of preparedness measures, including inventory check, securing supplies, and verifying the structural and functional integrity of equipment and infrastructure.
2. Impact window: The anticipated duration of disaster-related disruption informs the scale of required stockpiles and contingency measures.

Planning should be based on worst-case assumptions, ensuring sufficient resilience under strain.

##### Which?

Assessment of available resources is essential to estimate the unit’s maximum sustainable operational capacity during disruption. Critical elements include the following:

1. Dialysis machinery functionality.
2. Integrity of water systems (pipes and wiring), water storage capacity, and sources of raw water.
3. Availability of backup systems (e.g., generators and portable reverse osmosis units).
4. Supplies (dialysis consumables including gloves, gauze, puncture needles, dialysis tubing sets, dialyzers, and catheters), medications, and antiseptics.

Among medications, potassium-lowering agents are particularly crucial, because of the heightened risk of hyperkalemia-related complications during disasters.^36^ Adjunct therapies, such as loop diuretics, potassium-binders, sodium bicarbonate and phosphate-binders, and antihypertensives may serve as temporary supportive measures when dialysis access is limited.^37^ In scenarios of prolonged disruption, accommodating staff and patients on-site may be necessary, highlighting the importance of nonperishable food supplies.

##### Who?

Effective implementation requires clearly defined roles and responsibilities. Designated personnel should oversee coordination, scheduling adjustments, and clinical decision-making. Organizing and distributing responsibilities among multiple staff members can alleviate ethical burden and reduce emotional distress.^9^ Postdisaster evaluation of patients is also essential to identify new or evolving complications.

##### Where?

Geographical considerations influence both accessibility and safety. Disruptions to transportation may prevent patients and staff from reaching or leaving the unit. Coordination ahead of time with nearby facilities to share capacity can mitigate access barriers. Additionally, contingency plans should include criteria for patient evacuation when necessary. During a disaster, clinicians must also determine whether some patients require evacuation for life-saving interventions or emergent specialist evaluation. These issues should be factored into the disaster preparedness plan if possible.

##### How?

Operational continuity may be compromised by power instability and digital system failure. In such circumstances, reliance on low-technology solutions becomes critical. Maintaining paper-based patient summaries (Figure 1) ensures continuity of care when electronic medical records are inaccessible. These records should be regularly updated and incorporated into drills.

**Figure 1:**
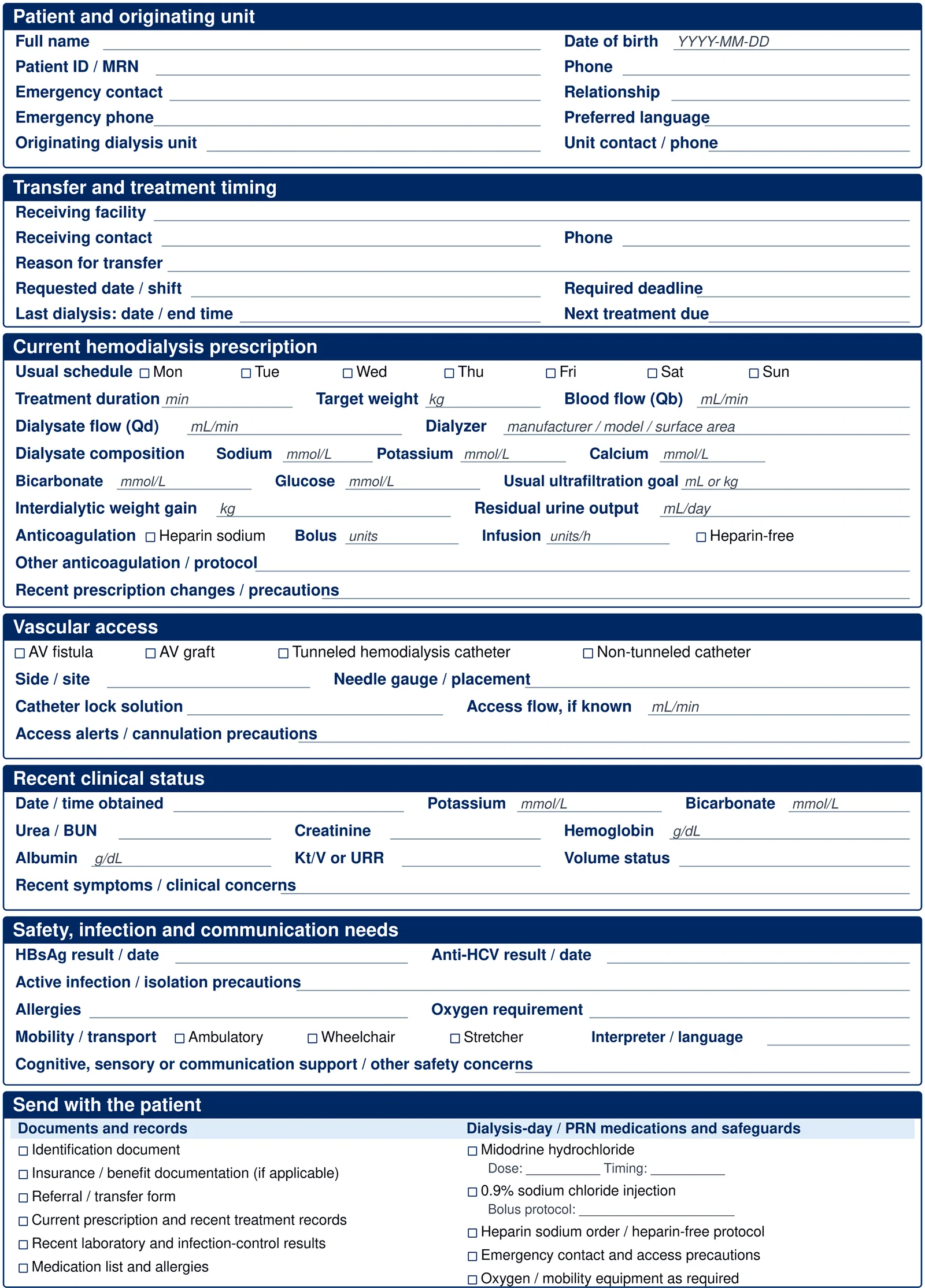
Emergency dialysis patient summary supporting continuity of care during digital or infrastructure disruption. This generalized template should be adapted to local documentation and regulatory requirements.

##### Scenario Summary and Implications

This simulation highlights a high-risk yet plausible scenario in which digital infrastructure failure and logistical disruption converge to threaten the continuity of dialysis care. It underscores the vulnerability of modern, technology-dependent health care systems when confronted with environmental and infrastructural instability. The “5W1H” framework facilitates a structured and comprehensive approach to disaster preparedness. Importantly, this scenario emphasizes that resilience extends beyond infrastructure to include patients and unit members. Furthermore, the need for operational redundancy, particularly through low-technology backups such as paper-based documentation, remains indispensable despite ongoing digital transformation. This scenario further advocates for regional collaboration, adaptable resource allocation, and preestablished contingency networks to mitigate disparities in access during crisis. However, the applicability of the 5W1H framework may vary according to disaster type. Although it provides a useful structure for preparedness planning in predictable or semi-predictable events such as typhoons, seasonal flooding, or forecastable heat waves, its use may be more limited during sudden-onset disasters characterized by rapid system failure (e.g., major earthquakes, armed conflicts, or cyberattacks).

#### Session 3: Future Directions for Resilient Kidney Care

Modern kidney replacement therapy remains highly dependent on centralized infrastructure systems that are increasingly vulnerable to disruption. The future of disaster nephrology will require both the strategies outlined above and a broader shift toward decentralized, adaptive dialysis models.^32^ Decentralized dialysis models may not only improve flexibility during disasters but also function as structural resilience mechanisms that reduce dependence on vulnerable centralized infrastructure.^1^, ^38^ Experience during the COVID-19 pandemic suggested that home-based dialysis may support treatment continuity and operational flexibility during large-scale disruptions.^39^ Accordingly, several recent disaster nephrology frameworks have increasingly emphasized distributed care systems, caregiver-assisted dialysis, and flexible treatment adaptation as components of long-term preparedness planning (Figure 2).^15^, ^32^ Home dialysis may decentralize kidney replacement therapy and reduce dependence on travel to a single facility, which may be advantageous when roads or centralized services are disrupted.^40^, ^41^ Peritoneal dialysis (PD) requires reliable delivery and safe storage of sterile solutions and the ability to switch from automated to manual exchanges when power is unavailable. Therefore, preparedness requires patient and care-partner training, modality-specific contingency prescriptions, medication and supply planning, backup communication, and a predetermined pathway to an operational facility when home treatment is unsafe or unavailable. Home dialysis patients should be included in regional registries, supply allocation, communication, evacuation, and shelter planning. Session participants noted that decentralized dialysis may be particularly valuable during conflicts and other disasters that cause prolonged disruption of infrastructure. For patients already receiving PD, the ability to switch from automated PD to manual exchanges may help maintain treatment when electricity or access to cyclers is disrupted. The panel also highlighted the potential role of PD in treating disaster-associated acute kidney injury. The International Society for Peritoneal Dialysis guideline recommends that PD be considered a suitable modality for acute kidney injury in all settings and provides minimum standards for resource-constrained circumstances.^42^ Commercially prepared solutions are preferred, but when they are unavailable, locally prepared solutions prepared with careful adherence to sterile procedures may be lifesaving.^43^ Therefore, acute PD may warrant inclusion in disaster preparedness plans, particularly when extracorporeal dialysis capacity or conventional water and power infrastructure is severely constrained.^44^, ^45^ Nevertheless, its implementation requires appropriate catheter access, aseptic procedures, trained personnel, adequate supplies, and clinical monitoring.^43^

**Figure 2:**
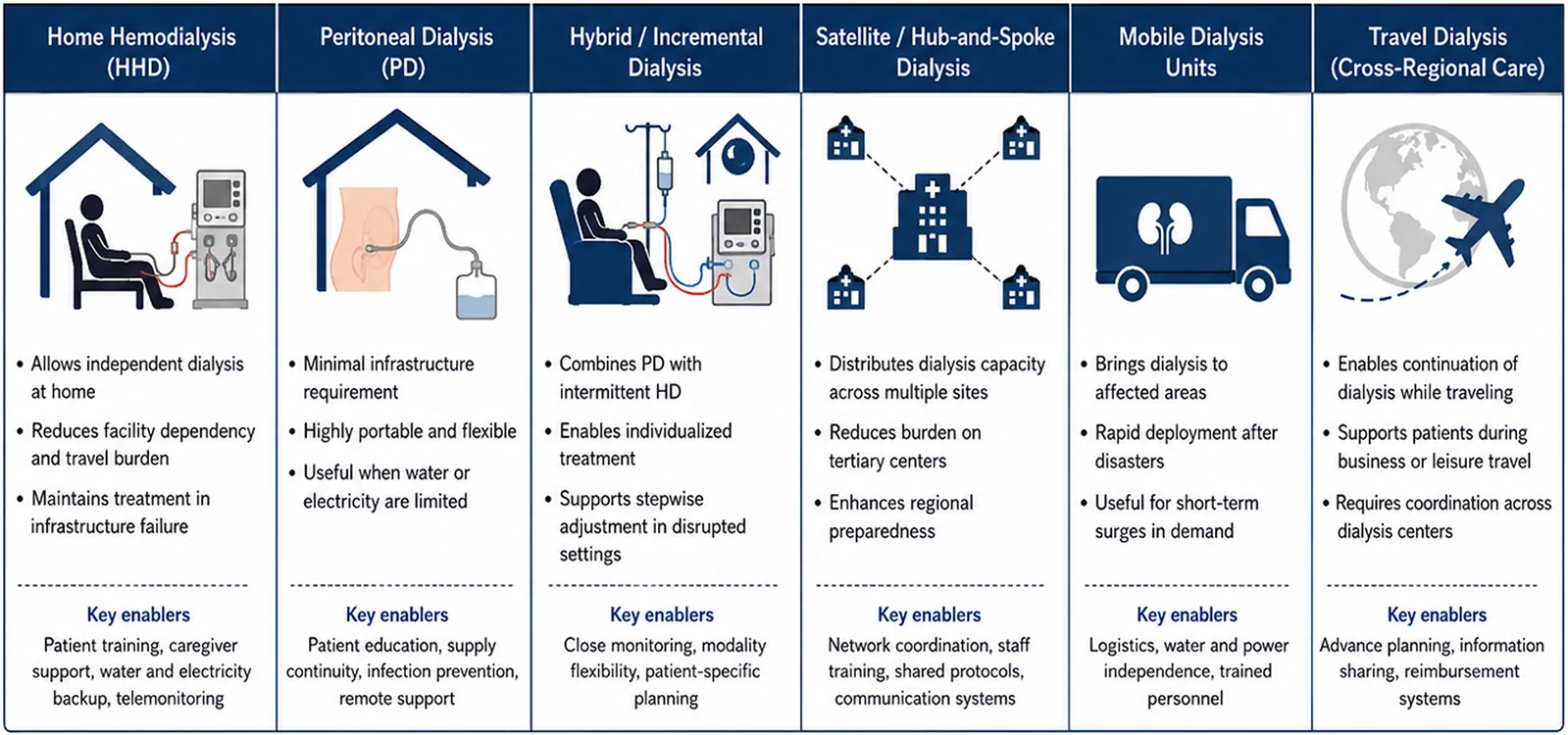
Resilience framework for maintaining continuity of kidney care during disasters.

AI was also discussed as a possible aid to anticipatory preparedness and operational coordination during large-scale disruptions. A systematic review identified potential applications of AI and machine learning in disasters and public health emergencies, including evacuation planning, rapid information processing, casualty assessment, and postdisaster decision support.^46^ In kidney care, hazard forecasts could in principle be combined with local data on dialysis demand and urgency, facility and infrastructure status, treatment schedules, transport accessibility, staffing, and supplies to identify emerging service gaps. This dialysis-specific application remains a forward-looking proposal and has not been clinically validated. Recent machine-learning weather models, including GenCast and NeuralGCM, have improved selected medium-range forecasts relevant to extreme weather, with forecast horizons extending up to 1 to 2 weeks.^47^, ^48^ Their usefulness in disaster nephrology will depend on data quality, connectivity, interoperability, representativeness, local validation, and maintenance. Therefore, AI-assisted systems should complement human decision-making and established low-technology contingency plans.^47^, ^48^ However, dependence on digital AI systems may also become a vulnerability during disasters.

By 2050, kidney care may face more frequent compound climate-related disruptions, population aging, rising dialysis demand, displacement, and greater reliance on interconnected digital systems. Decentralized care, regional supply networks, and AI-assisted forecasting may strengthen continuity but could also introduce risks related to inequitable access, cybersecurity, power, and connectivity. Therefore, future innovations should be paired with locally maintainable capacity and low-technology backup pathways. Importantly, resilience should not be viewed solely as an infrastructure issue. Patients receiving dialysis frequently experience psychological stress,^49^ social isolation, transportation insecurity,^22^ and medication interruption during disasters.^35^ Pediatric patients and socially vulnerable populations may face additional challenges related to caregiver dependency and evacuation logistics.^50^ Therefore, future preparedness strategies should integrate patient education, caregiver engagement, psychosocial support, and community-level coordination into broader resilience planning. Importantly, disaster preparedness in nephrology should also extend beyond dialysis populations to include patients with nondialysis chronic kidney disease, who may experience worsening hypertension, volume overload, hyperkalemia, medication interruption, dehydration, and delayed access to laboratory monitoring during disasters.^51^ Potential preparedness strategies may include maintaining updated medication lists, preserving limited medication reserves when feasible, establishing backup follow-up pathways, and educating patients regarding hydration safety and emergency dietary modification (Supplementary Table S1).

Building on the session discussion and published literature, the participants identified multilevel governance as an important link between preparedness planning and operational resilience. At the facility level, internal command structures and standardized reporting can support timely decision-making. Regional coordination centers can map operational capacity, match patients with available treatment slots, and coordinate transport and supplies. National and cross-border networks may facilitate mutual aid when local capacity is exhausted. Standardized minimum data sets, communication protocols, and low-technology backup channels can also help maintain situational awareness when routine digital systems fail.^10^, ^11^ The discussion also underscored that preparedness must be adapted to baseline capacity.^32^ The same hazard may have very different consequences in settings with redundant networks and in those where dialysis capacity is already limited before a disaster. Portable reverse osmosis systems, telemedicine, large stockpiles, and rapid regional transfer may not be feasible in every setting. Therefore, a resource-stratified approach may begin by protecting locally maintainable minimum functions. These include an up-to-date patient registry, basic urgency and referral criteria, paper-based or locally accessible patient records, redundant contact methods, practical agreements with the nearest functioning facilities, patient education on emergency diet and fluid restriction, and realistic plans for interruptions in transport, water, power, staffing, and supplies. Additional components may be added according to local infrastructure, workforce, financing, and maintenance capacity.^15^ International and cross-border support can strengthen locally led systems rather than assume that technology-intensive models are universally available.^11^, ^17^ These considerations may also inform the planned development of the International Society of Nephrology Renal Disaster Preparedness Working Group disaster preparedness toolkit. The proposed toolkit aims to translate lessons from the session and subsequent work into adaptable workflows, checklists, minimum data sets, and triage and communication tools. It is intended to support locally led implementation and clarify when regional or international assistance may be needed, rather than prescribe a single technology-dependent model.

## Conclusion

The session discussion and subsequent author synthesis suggest that disaster nephrology is evolving beyond traditional acute-response paradigms toward long-term preparedness and resilience. This preparedness should occur at multiple levels and involve health care systems, facilities, and individuals, including patients, with reinforcement of both physical and psychological resilience. Participants also emphasized that preparedness frameworks should remain adaptable to different disaster contexts, recognizing that operational priorities may differ between predictable hazards, such as typhoons and floods, and sudden-onset events, including earthquakes, armed conflicts, and large-scale infrastructure failures. Future efforts should integrate resilient infrastructure, decentralized models of kidney care, and international collaboration to sustain continuity of care during increasingly frequent disasters and foster solidarity between variable resource settings.

## Disclosure

AAA, ST, SK, MW and KDY are all members of the International Society of Nephrology Renal Disaster Preparedness Working Group. The rest of the authors have no disclosures to report in relationship to this manuscript.

## Supplementary Material

Supplementary File (PDF)

**Table S1.** Key concerns and potential disaster planning strategies among patients with non-dialysis CKD.
