# Supplementary material for: When Disasters Are the Norm: A 2050 Kidney Care Simulation. Report of a World Congress of Nephrology 2026 Spotlight Session
Source: Kidney Int Rep. 2026 Sep 25;11(12):107092. doi: 10.1016/j.ekir.2026.107092 (PMC13744217; doi:10.1016/j.ekir.2026.107092)
Supplement: Supplementary file 1 [file mmc1.pdf]

Table S1. Key concerns and potential disaster planning strategies among patients with non-dialysis CKD

Key concerns	Why matters	Strategies
Medication continuity	Interruption of antihypertensives, anti-diabetic medications, diuretics, potassium and phosphate binders, sodium bicarbonate, or anemia-related medications, may de-stabilize CKD.<br><br>Interruption of anti-rejection medications may increase risk of renal allograft rejection and dysfunction.	1. Keep an updated medication list<br>2. Maintain a 1–2-week reserve (if feasible), periodically check expiration date<br>3. Store medications in waterproof packaging<br>4. Identify alternative pharmacies<br>5. Review sick-day rules with primary physicians
Blood pressure and volume control	Disaster may alter diet, fluid intake, temperature exposure, and medication access, the combination of which can worsen hypertension, edema, or on the contrary, dehydration	1. Prepare a home blood pressure monitoring machine<br>2. Record usual target blood pressure and comfortable weight<br>3. Clarify when to hold diuretics, RAS blockers, or SGLT2 inhibitors if dehydration becomes a concern, with primary physicians<br>4. Identify symptoms of hypotension requiring urgent care by oneself
Diabetes management	Food disruption, infection, stress, and medication interruption can worsen glycemic control and precipitate acute kidney injury	1. Prepare a home glucometer and strips, insulins or anti-diabetic medications if appropriate<br>2. Prepare snacks for emergent use<br>3. Identify symptoms of hypoglycemia requiring urgent care by oneself

Key concerns	Why matters	Strategies
Hyperkalemia risk	Limited food access may increase reliance on high-potassium shelf-stable foods; in addition, delayed laboratory tests may miss rising serum potassium levels	1. Prepare kidney-friendly emergency food options<br>2. Keep a low-potassium food list<br>3. Ensure potassium binders available<br>4. Identify symptoms of hyperkalemia requiring urgent care by oneself
Acute kidney injury on chronic kidney disease	Heat, dehydration, infection, analgesic use, and delayed care can all precipitate acute kidney injury	1. Emphasize hydration safety<br>2. Avoidance of NSAIDs and nephrotoxins<br>3. Self-care for infections or diarrhea, and clear understanding about when to seek emergent care
Dietary restrictions	Emergency food supplies are often high in sodium, potassium, phosphorus, and sugar	1. Procure a CKD-adapted disaster food list, such as low-sodium canned foods, low potassium fruit, kidney-appropriate snacks, bottled water, and phosphate-conscious choices
Laboratory and follow up disruption	CKD management depends on periodic monitoring of kidney function, electrolytes, bicarbonate, and hemoglobin	1. Identify back-up clinics or laboratories<br>2. Arrange telemedicine or phone-based follow-up when available
Psychosocial stress	Disasters can worsen anxiety, depression, sleep disruption, and adherence	1. Screen for social isolation<br>2. Provide mental health resources and community support if available
